# Premature ejaculation and stress

**DOI:** 10.1192/j.eurpsy.2022.723

**Published:** 2022-09-01

**Authors:** R. Sajdlova, L. Fiala

**Affiliations:** University Hospital in Pislen, Psychiatry Department, Pilsen, Czech Republic

**Keywords:** premature ejaculation, Stress, Cortisol

## Abstract

**Introduction:**

Recent findings indicate that men with premature ejaculation report more frequent sexual problems associated with increased anxiety and interpersonal difficulties. Also the neuroendocrine changes were examined and compared to other indicators of stressful experiences.

**Objectives:**

Premature Ejaculation (PE) is defined as an ejaculation occurring within one minute after the start of sexual intercourse and occurs in 20-30% of men. They report frequent problems with partnerships and increased anxiety, irritability and orgasmic dysfunction. Premature ejaculation is likely to be associated with decreased serotonergic neurotransmission and higher levels of leptin. Also the role of hyperactive thyroid and prostate disease was investigated. On the other hand there is no evidence as to how previous stressful experience and distrubed partnership might contribute PE.

**Methods:**

Our study comprised 60 male outpatients diagnosed as having secondary premature ejaculation. Clinical examinations were focused on biochemical analysis of cortisol and psychometric scoring using a diagnostic tool for premature ejaculation, traumatic stress and somatoform dissociation. The control group consisted of a 60 healthy men.

**Results:**

The results showed significant Spearman correlations of the Premature Ejaculation Diagnostic Tool score with Trauma symptoms checklist score (R=0.86), cortisol level (R=0.47) and Somatoform dissociation questionnaire score (R=0.61). In the control group, the results did not reach statistical significance. Spearman correlations of the Premature Ejaculation Diagnostic Tool score with Trauma symptoms checklist score was (R=0.21), cortisol (R=0.27) and with Somatoform dissociation questionnaire score (R=0.25).

**Conclusions:**

These results represent the first reported findings documenting the relationship of traumatic stress indicators with the experience of secondary premature ejaculation and cortisol levels.

**Disclosure:**

No significant relationships.

